# Best evidence summary on gastrointestinal function recovery after radical cystectomy for bladder cancer

**DOI:** 10.1016/j.apjon.2025.100824

**Published:** 2025-11-30

**Authors:** Yumei Dai, Xiaoju Zhang, Qianwen Lin, Xiaofeng Gu

**Affiliations:** a Department of Nursing, Fudan University Shanghai Cancer Center, Shanghai 200032, China; b Department of Oncology, Shanghai Medical College, Fudan University, Shanghai 200032, China

**Keywords:** Gastrointestinal function, Bladder cancer, Radical cystectomy, Evidence summary

## Abstract

**Objective:**

To summarize the best available evidence on gastrointestinal function recovery in patients with bladder cancer undergoing radical cystectomy.

**Methods:**

A systematic search was conducted following the Evidence-Based Health care Pyramid 5.0, covering major academic databases, professional society websites, and clinical decision support tools. Literature published up to March 31, 2025, was included. Two independent reviewers assessed the quality of included studies and extracted relevant evidence.

**Results:**

Nine articles were included, comprising four clinical guidelines and five expert consensuses. The overall methodological quality was high. A total of 18 evidence items were identified, spanning the preoperative, intraoperative, and postoperative phases of care.

**Conclusions:**

This review identified 18 evidence-based recommendations for gastrointestinal recovery after radical cystectomy by integrating Enhanced Recovery After Surgery concepts with traditional Chinese medicine techniques. These findings offer practical references for nursing care, while future studies are needed to support evidence implementation and improve outcomes.

**Systematic review registration:**

This study was registered in the Fudan University Centre for Evidence-based Nursing (Registration No. ES20257819).

## Introduction

Bladder cancer is the ninth most common cancer worldwide, with approximately 614,000 new cases reported in 2022, over three-quarters of which occurred in males.^1^ Approximately 20% to 25% of patients are diagnosed with muscle-invasive bladder cancer (MIBC) at initial presentation, which is characterized by aggressive tumor behavior and poor survival outcomes.^2^^,^^3^ The primary treatment for MIBC patients is radical cystectomy (RC). RC is a complex procedure that often requires the resection of intestinal segments and multiple reconstructive anastomoses for urinary diversion.^4^ As a result, the incidence of postoperative gastrointestinal dysfunction (PGD) is relatively high, accounting for 10% to 30%.^5^

PGD is a common complication after anesthesia and surgery, characterized by symptoms such as nausea, vomiting, abdominal pain, bloating, altered bowel movements, reduced bowel sounds, impaired gut motility, and delayed flatus or defecation.^6^ When PGD occurs, it can delay patients' recovery, prolong the length of stay, and increase the overall health care burden for both patients and the medical system.^7^ Therefore, implementing gastrointestinal (GI) management during the perioperative period is essential for supporting GI recovery in patients undergoing RC.

Although some guidelines or recommendations have addressed GI function recovery in RC, relevant evidence is often embedded within broader perioperative care frameworks.^8^^,^^9^ As a result, focused discussion and systematic evidence specific to GI recovery remain limited. Moreover, most available evidence on GI recovery originates from GI surgeries. However, since RC involves both the urinary and digestive systems, it remains uncertain whether such evidence is directly applicable to this population. In recent years, traditional Chinese medicine (TCM), including acupoint stimulation and herbal therapies, has been increasingly explored as a complementary approach in perioperative care. Although the integration of TCM into routine postoperative care varies across institutions, TCM-based interventions are widely used in Chinese clinical practice and have demonstrated potential benefits in managing symptoms like nausea, pain, and impaired gastrointestinal function.^10^, ^11^, ^12^ However, most of the current evidence focuses on patients undergoing abdominal or thoracic surgeries, few studies have explored the integration of TCM into GI recovery strategies for patients undergoing RC.

Therefore, this study aims to synthesize evidence from Enhanced Recovery After Surgery (ERAS) concepts and incorporate insights from TCM to develop a more tailored approach for postoperative GI recovery in patients undergoing RC.

## Methods

### Clinical question formulation

The clinical question was developed using the PIPOST framework, which was identified as follows: P (Population): Bladder cancer patients with radical cystectomy; I (Intervention): included but not limited to ERAS strategies, early enteral nutrition, probiotics, prokinetic agents, gum chewing, and acupoint stimulation. P (Professional): nurses, clinicians, caregivers, and anesthesiologists. O (Outcome): the incidence of PGD. S (Setting): the urology ward. T (Study type): evidence summaries, guidelines, recommendations, consensus, systematic reviews, and meta-analysis.

### Sources of evidence and search strategy

The search followed the top-down principle of the practising evidence-based health care pyramid 5.0.^13^ We searched international databases such as BMJ Best Practice, UpToDate, ERAS Society, European Association of Urology (EAU), American Urological Association (AUA), National Institute for Health and Care Excellence(NICE), Guidelines International Network (GIN), Cochrane Library, PubMed, ScienceDirect, Web of Science, Scopus, Embase, and Google scholar. The Chinese databases included China Biology Medicine (CBM), China National Knowledge Infrastructure (CNKI), Wanfang Data, and China Science and Technology Journal Database (VIP). We additionally searched Chinese clinical and policy websites, including Medlive and the National Health Commission (NHC), to supplement guideline and expert consensus retrieval.

The search strategy employed a combination of subject headings and free-text terms. Studies published from the inception of each database through March 31, 2025, were included. The complete PubMed search strategy is presented in Table 1. Strategies for other databases were adapted accordingly.

**Table 1:** Search strategy of Pubmed.

Step	Search items
#1	"Cystectomy"[Mesh] OR "urinary Diversion"[Mesh] OR (bladder Neoplasms[Mesh] AND Surgery[Subheading]) OR radical Cystectomy[Title/Abstract] OR bladder cancer Surgery[Title/Abstract] OR urinary Diversion[Title/Abstract]
#2	"Gastrointestinal Motility"[Mesh] OR "Ileus"[Mesh] OR "gastrointestinal Diseases"[Mesh] OR gastrointestinal Recovery[Title/Abstract] OR postoperative Ileus[Title/Abstract] OR bowel Function[Title/Abstract] OR gut Microbiota[Title/Abstract] OR″Enhanced recovery after Surgery"[Mesh] OR "enteral Nutrition"[Mesh] OR "Probiotics"[Mesh] OR "analgesia, Epidural"[Mesh] OR "fluid Therapy"[Mesh] OR ERAS[Title/Abstract] OR Fast-track Surgery[Title/Abstract] OR chewing Gum[Title/Abstract] OR prokinetic Agents[Title/Abstract] OR preoperative carbohydrate Loading[Title/Abstract] OR fluid Management[Title/Abstract] OR opioid Sparing[Title/Abstract] OR Epidural [Title/Abstract] OR "medicine, Chinese Traditional"[Mesh] OR "transcutaneous electrical acupoint Stimulation"[Title/Abstract] OR "Electroacupuncture"[Title/Abstract] OR "Acupuncture"[Mesh] OR "auricular pressing"[Title/Abstract] OR "acupoint Application"[Title/Abstract]
#3	Systematic review[Title/Abstract] OR meta analysis[Title/Abstract] OR Guideline[Title/Abstract] OR Recommendation[Title/Abstract] OR Consensus[Title/Abstract] OR evidence summary[Title/Abstract]
#4	#1 AND #2 AND #3

### Inclusion and exclusion criteria

The inclusion criteria for literature selection were as follows: (1) studies involving patients with bladder cancer who underwent RC; (2) research focusing on GI function recovery or ERAS; (3) publications written in English or Chinese; (4) research types limited to clinical guidelines, expert consensus or recommendations, systematic reviews, meta-analysis, or best evidence summaries. Exclusion criteria included (1) unavailability of full text; (2) interventions or outcomes were not aligned with the research topic; (3) and records were repeatedly indexed in different databases.

### Critical appraisal

Two qualified critical appraisers assessed the literature individually. An expert was consulted when critical appraisers expressed different opinions. The three reviewers then discussed the differences to reach a consensus. If consensus could not be reached through discussion, the final decision was made by majority vote. Guidelines were assessed by the Appraisal of Guidelines for Research and Evaluation (AGREE Ⅱ) tool.^14^ Expert consensus or recommendations were assessed using the Joanna Briggs Institute (JBI) Critical Appraisal Checklist for text and opinion.^15^ Systematic review and meta-analysis were assessed using the JBI Critical Appraisal Checklist for systematic reviews and research syntheses.^16^ Evidence summaries were assessed by tracing the original studies and conducting a quality appraisal according to the study types.^17^ Inter-rater reliability was assessed using the intraclass correlation coefficient (ICC). An ICC value ≥ 0.75 indicated good agreement, and ≥ 0.90 excellent agreement.^18^

### Evidence extraction and recommendation level

Evidence was extracted from the literature initially retrieved and assessed for quality. The extracted evidence was then organized into thematic categories based on content. We used the JBI Evidence Pre-Grading and Evidence Recommendation System (2014 version) to assess the quality of evidence extracted from the literature.^17^ Based on different study designs, the evidence was traced back to the original references and classified into Grades 1 to 5. The recommendation level of the evidence was classified as Grade A or Grade B using the feasibility, appropriateness, meaningfulness, and effectiveness (FAME) framework.^19^

## Results

**Fig. 1:**
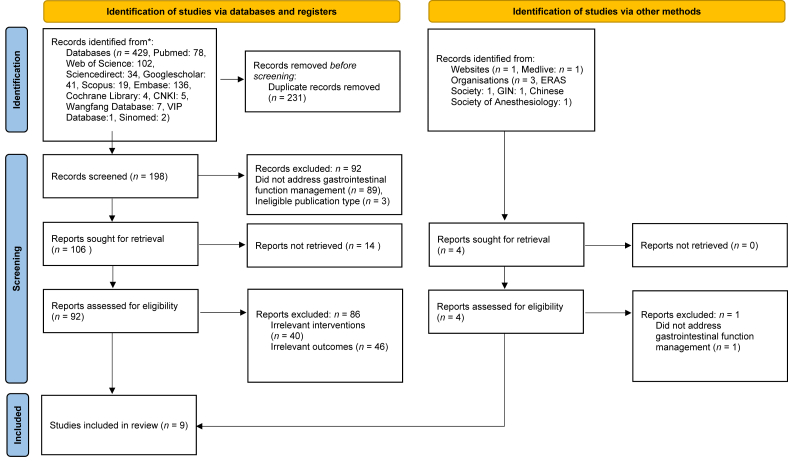
Flowchart of literature selection process.

A total of 433 records were initially identified. After duplicate removal using EndNote X9 (Clarivate) and subsequent title and abstract screening conducted independently by two reviewers, 9 studies were ultimately included (Fig. 1). Among them, 4 were guidelines and 5 were expert consensus documents. Detailed information is presented in Table 2.

**Table 2:** Characteristics of included articles.

Author	Year	Article type	Article source	Topic
Xue et al.^6^	2024	Guideline	Medlive	Integrated Chinese and Western medicine strategies for the prevention and management of postoperative gastrointestinal dysfunction
Cerantola et al.^8^	2013	Guideline	ERAS society	Perioperative care strategies based on enhanced recovery after surgery (ERAS) for patients undergoing radical cystectomy for bladder cancer
Witjes et al.^20^	2021	Guideline	Sciencedirect	Guidelines for the diagnosis, treatment, and perioperative management of muscle-invasive and metastatic bladder cancer
Roumiguié et al.^21^	2024	Guideline	Embase	Updated guidelines from the French Urological Association on the management of muscle-invasive bladder cancer
Albisinni et al.^22^	2025	Expert consensuses	PubMed	Ten-year review and surgeons' recommendations on enhanced recovery after surgery (ERAS) for patients undergoing radical cystectomy
Collins et al.^23^	2015	Expert consensuses	PubMed	Consensus and best practices on ERAS in robot-assisted radical cystectomy
ERAS-BC Consortium^9^	2018	Expert consensuses	Wangfang	Expert consensus on enhanced recovery after surgery for radical cystectomy and urinary diversion
Chinese Urological surgery professional Committee of Chinese research hospital Association^24^	2021	Expert consensuses	Wangfang	Consensus on perioperative safety management in patients undergoing radical cystectomy with urinary diversion
Wang et al.^25^	2021	Expert consensuses	Chinese society of Anesthesiology	Expert consensus on acupoint stimulation for the prevention and management of postoperative gastrointestinal dysfunction

ERAS-BC, Enhanced Recovery After Surgery-bladder cancer.

### Quality evaluation of literature

#### Quality evaluation of guidelines

This study included four guidelines, which were assessed by AGREE Ⅱ. Three guidelines scored above 60% in all domains and were rated Grade A. One guideline scored 56.25% in Applicability but exceeded 60% in the remaining domains, resulting in a Grade B rating (Table 3). All intraclass correlation coefficient (ICC) values for guideline ratings were ≥ 0.75, indicating good inter-rater reliability.

**Table 3:** Quality evaluation of guidelines (*N* = 4).

Standard scores of guidelines (%)										
Author	Scope and purpose	Stakeholder involvement	Rigour of development	Clarity of presentation	Applicability	Editorial independence	≥ 60%	≥ 30%	ICC	Quality evaluation
Xue et al.^6^	94.44	100.00	84.38	91.67	56.25	91.67	5	6	0.81	B
Cerantola et al.^8^	100.00	66.67	88.54	94.44	64.58	95.83	6	6	0.94	A
Witjes et al.^20^	100.0	83.33	98.96	100.00	60.42	95.83	6	6	0.95	A
Roumiguié et al.^21^	100.00	83.33	78.13	100.00	70.83	66.67	6	6	0.93	A

ICC, intraclass correlation coefficient.

#### Quality evaluation of expert consensus statements

A total of five expert consensus statements were included. Of these, two met all appraisal items with a "Yes" rating, while three were rated "Unclear" for the final item only (Table 4). Overall, the methodological quality of the literature was high.

**Table 4:** Quality evaluation of expert consensuses (*N =* 5).

Author	Item 1	Item 2	Item 3	Item 4	Item 5	Item 6
Albisinni et al.^22^	YES	YES	YES	YES	YES	YES
Collins et al.^23^	YES	YES	YES	YES	YES	Unclear
ERAS-BC Consortium et al.^9^	YES	YES	YES	YES	YES	Unclear
Chinese Urological surgery professional Committee of Chinese research hospital Association^24^	YES	YES	YES	YES	YES	Unclear
Wang et al.^25^	YES	YES	YES	YES	YES	YES

ERAS-BC, Enhanced Recovery After Surgery-bladder cancer; JBI, Joanna Briggs Institute. The quality evaluation was assessed by the JBI critical appraisal checklist for textual evidence: expert opinion. Item 1: Is the source of the opinion clearly identified? Item 2: Does the source of opinion have standing in the field of expertise? Item 3: Are the interests of the relevant population the central focus of the opinion? Item 4: Does the opinion demonstrate a logically defended argument to support the conclusions drawn? Item 5: Is there reference to the extant literature? Item 6: Is any incongruence with the literature/sources logically defended?

### Summary and description of evidence

This study summarized 18 items of evidence from the preoperative, intraoperative, and postoperative periods. Preoperative management include nutritional support, bowel preparation, fasting and carbohydrate loading, and TCM-based techniques. The intraoperative period consisted of fluid management and surgical technique selection. The postoperative management mainly included pharmacological therapy, nutritional management, TCM interventions, early mobilization, tube and drain management, and structured follow-up care (Table 5)

**Table 5:** Summary and description of evidence.

Topic	Evidence content	Level of evidence	Recommendation level
**Preoperative management**	1. Nutritional support: Preoperative nutritional support is recommended. For those with severe malnutrition, nutritional support should be provided 7–10 days prior to surgery.^8^^,^^9^	Level 3	B
	2. Bowel preparation: Routine preoperative mechanical bowel preparation and oral antibiotics are not recommended.^8^^,^^22^^,^^23^	Level 1	A
	3. Preoperative fasting and carbohydrate loading: Fasting is recommended for 6 hours, and no drinking for 2 hours before surgery. Non-diabetic and diabetic patients with well-controlled blood glucose may ingest a clear carbohydrate-rich drink (e.g., glucose or maltodextrin solution) 2–3 hours preoperatively. Vegetables should be avoided within 24 hours before surgery.^9^^,^^21^,^22^^,^^23^	Level 1	A
	4. Emotional Regulation with traditional Chinese medicine (TCM): TCM-based external therapies such as acupuncture, acupressure, auricular therapy, electroacupuncture, and music therapy are recommended to alleviate preoperative anxiety and fear, thereby promoting postoperative gastrointestinal recovery.^6^	Level 1	B
	5. Preoperative acupoint stimulation: Preoperative use of acupoint stimulation techniques, including transcutaneous electrical acupoint stimulation (TEAS), electroacupuncture, auricular pressing, or application of acupoint patches, is recommended to enhance postoperative gastrointestinal recovery. Recommended acupoints include: Body acupoints: ST36 (Zusanli), PC6 (Neiguan), ST37 (Shangjuxu). Auricular acupoints: Shenmen, Sympathetic, Stomach, Spleen TEAS or electroacupuncture should be applied for 30 minutes before anesthesia induction. Auricular pressing and acupoint patches may be initiated one day before surgery and continued for 2–3 days postoperatively.^6^	Level 1	A
**Intraoperative management**	6. Fluid management: Intraoperative fluid administration should be limited to physiological needs while maintaining hemodynamic stability (≤ 5 mL/kg/h). This approach helps reduce the surgical stress response and tissue edema, promoting recovery of gastrointestinal function.^8^^,^^9^^,^^22^^,^^23^	Level 1	A
	7. Surgical technique selection: Minimally invasive techniques (e.g., laparoscopic or robot-assisted radical cystectomy) are recommended to reduce the systemic inflammatory response, surgical stress, and the risk of postoperative ileus.^8^^,^^9^^,^^22^^,^^23^	Level 1	A
**Postoperative management**	8. Nasogastric Intubation: Routine placement of nasogastric tubes pre- or postoperatively is not recommended for patients undergoing radical cystectomy.^9^	Level 2	A
	9. Postoperative analgesia: A multimodal opioid-sparing analgesia strategy is recommended, including Non-Steroidal Anti-inflammatory Drugs (NSAIDs), local wound infiltration with ropivacaine, neuraxial techniques, and nerve blocks. For patients undergoing laparoscopic surgery, oral analgesics are suitable following early oral intake. Routine use of epidural analgesia is not recommended after laparoscopy.^9^^,^^20^^,^^22^^,^^23^	Level 2	A
	10. Pharmacologic intervention for postoperative nausea and vomiting (PONV): Administer antiemetic agents regularly, such as metoclopramide, to control PONV.^20^^,^^23^	Level 2	A
	11.Acupoint stimulation techniques for PONV: Electroacupuncture, TEAS, manual acupuncture, auricular pressing, acupressure, or their combinations are recommended.^6^	Level 1	A
	12. Sham feeding: From postoperative day 1, chew gum at least thrice daily until bowel motility is restored.^8^^,^^20^^,^^21^^,^^22^^,^^23^	Level 1	A
	13. Nutritional management: Encourage early oral feeding. Actively correct hypoproteinemia and maintain electrolyte homeostasis, particularly potassium and sodium balance.^8^^,^^23^^,^^24^	Level 1	A
	14. Postoperative acupoint stimulation: Acupoint stimulation is recommended starting from postoperative day one, applied to bilateral Zusanli (ST36), Shangjuxu (ST37), Sanyinjiao (SP6), and Hegu (LI4) for 30 minutes per session, continued for 3 days, to enhance gastrointestinal recovery.^25^	Level 1	A
	15. Chinese herbal medicine for postoperative Paralytic ileus: In patients with postoperative paralytic ileus following abdominal surgery, it is recommended to combine conventional Western medical therapy with oral administration of modified traditional formulas such as Dachengqi Decoction, Xiaochengqi Decoction, Simotang, or Tongfu Decoction.^6^	Level 4	B
	16. Early mobilization: Once patients regain consciousness postoperatively, initiate a semi-recumbent position or in-bed movement. Routine 6-h flat-bed rest is not required.^9^ Encourage bedside standing or chair sitting within 24 hours after surgery. From postoperative day 2 onward, ambulation begins. Set daily activity goals and progressively increase activity volume.^6^^,^^21^^,^^23^	Level 1	A
	17. Ureteral Stent Drainage for Orthotopic Neobladder: For patients undergoing orthotopic neobladder reconstruction, external drainage of ureteral stents is recommended to improve upper urinary tract drainage and promote gastrointestinal recovery.^9^	Level 1	A
	18. Postoperative follow-up: A quantifiable and operable follow-up protocol should be developed under patient safety. Conduct a telephone follow-up 24–48 hours after discharge to assess recovery. Maintain close follow-up within the first postoperative month and establish a fast-track readmission pathway.^9^^,^^23^	Level 5	A2

## Discussion

### Main findings

This study focused on GI function recovery for bladder cancer patients undergoing RC. Building upon existing evidence-based practices, it incorporates both conventional and TCM interventions to develop a more comprehensive best-evidence set. The 18 included evidence items were categorized into three perioperative domains: preoperative optimization, intraoperative protection, and postoperative support. These domains provide a time-structured framework to guide clinical practice across the RC. Among the 18 included items, 12 (66.7%) were supported by Level 1 evidence and 15 (83.3%) were rated as Grade A recommendations, reflecting an overall high level of evidence quality. A few items, such as those involving Chinese herbal medicine and follow-up protocols, were based on lower-level evidence (Levels 4 to 5), suggesting the need for further high-quality research to strengthen clinical confidence.

#### Preoperative optimization: preparing body and mind for surgery

Items 1–5 summarize preoperative optimization for RC patients. These include nutritional support, bowel preparation, fasting and carbohydrate loading, and TCM-based techniques. RC can cause serious breakdown of body tissues, with large protein losses, fat burning, and ongoing weight loss.^26^ Therefore, preoperative nutritional support is vital in reducing postoperative complications and improving outcomes in RC patients.^27^ Recently, immunonutrition has emerged as a preferred approach during the RC perioperative period. This strategy combines standard oral nutritional formulations with specific immune-modulating nutrients, such as arginine, nucleotides, and fatty acids. It can enhance immune function, reduce inflammatory responses, and promote recovery in the catabolic state associated with surgery.^28^^,^^29^ There are mainly two nutritional support methods currently used in clinical practice, including enteral nutrition, and parenteral nutrition. Enteral nutrition, delivered orally or through tube feeding, can provide adequate caloric intake, support the gut barrier, and improve immune function, while being well tolerated. Parenteral nutrition can provide precise nutritional support through specific nutrient mixtures while avoiding malabsorption.^26^ Each route has its advantages and indications, but physiologically enteral nutrition should be prioritized preoperatively in RC patients based on their nutritional status and tolerance, with immunonutrition considered when necessary to improve prognosis.^30^ For those at risk of malnutrition before surgery, nutritional intervention should ideally begin 7–10 days in advance.^22^

Regarding bowel preparation, high-level international evidence does not support mechanical bowel preparation before RC, as its omission has not been shown to increase risks related to safety or postoperative complications.^8^^,^^22^^,^^23^ However, we found that the Chinese consensus still supports the use of limited bowel preparation with oral laxatives prior to surgery.^9^ Differences in clinical habits and cultural contexts may explain this divergence. Based on the current evidence, we recommend omitting routine bowel preparation before RC, with decisions tailored to individual patient conditions where appropriate.

As for the fasting time, the ERAS framework shortens preoperative fasting in RC patients to 6 hours for solids and 2 hours for clear liquids, as gastric emptying for fluids typically takes 60–90 minutes. This approach maintains safety by avoiding increased anesthesia-related aspiration risk while reducing fasting-related hunger and thirst to improve patient comfort.^31^ Preoperative carbohydrate loading helps mitigate glycemic fluctuations caused by surgery-induced insulin resistance. It also alleviates postoperative discomfort such as hunger, nausea, and vomiting, supporting GI recovery.^32^^,^^33^ Therefore, for patients without diabetes or with well-controlled blood glucose, it is recommended to consume carbohydrate-rich drinks 2–3 hours before surgery. However, despite its clinical benefits, the implementation of carbohydrate loading remains limited.^34^ Further efforts are required to enhance awareness and adoption in routine practice.

#### Intraoperative protection: minimizing gastrointestinal stress

Items 6–7 highlight the GI function protection strategies during RC, including the fluid management and surgical techniques. Fluid management aims to reach zero fluid balance during surgery. Studies have shown that fluid intake during surgery is U-shaped associated with postoperative complications, meaning that both insufficient and excessive fluid administration can be harmful to patients.^35^ However, RC is a complex surgery usually lasting more than 6 hours.^36^ The liberal fluid administration is common in practice, leading to excessive blood volume. This increases the release of atrial natriuretic peptide from the heart, contributing to the breakdown of the endothelial glycocalyx. As a result, the vascular barrier is compromised, leading to interstitial fluid accumulation, bowel edema, and delayed recovery of GI function.^37^ Therefore, it is vital to restrict fluid intake intraoperatively. The suggested volume of intraoperative intravenous fluids should not exceed 5 mL/kg/h^34^.

As for the choice of surgical approach, the minimally invasive surgery, such as robot-assisted radical cystectomy and laparoscopic radical cystectomy, is recommended for RC patients. Compared with an open procedure, minimally invasive surgery is performed within a sealed environment, shortening the duration of bowel exposure. Additionally, precise surgical instruments reduce intestinal traction and mechanical irritation. As a result, GI function tends to recover more quickly following minimally invasive procedures.^38^ However, minimally invasive surgery, particularly robot-assisted procedures, still faces challenges, including a steep learning curve, high costs, and a lack of high-quality evidence supporting its benefits in overall and recurrence-free survival.^39^, ^40^, ^41^ These limitations may hinder its broader clinical adoption. In the future, as RC techniques continue to evolve and their clinical application expands, well-designed, multicenter, prospective studies will be necessary to validate the cost-effectiveness and long-term oncological outcomes. These findings will help provide stronger evidence for clinical decision-making.

#### Postoperative support: facilitating gastrointestinal recovery

Items 8–17 outline the postoperative strategies for RC patients' GI recovery, covering pharmacological therapy, nutritional management, TCM interventions, early mobilization, tube and drain management, and structured follow-up care. Pharmacologic therapy is an essential component of postoperative GI recovery following RC. One widely recommended approach is the opioid-sparing multimodal analgesia strategy, which effectively controls postoperative pain while minimizing GI-related adverse effects such as ileus, nausea, and vomiting.^23^ This benefit is primarily attributed to the reduced use of opioids, which are known to inhibit GI motility by acting on μ-opioid receptors in the gut, leading to increased muscle tone and slowed intestinal motility.^42^ In addition, antiemetic agents such as metoclopramide are commonly used to prevent postoperative nausea and vomiting (PONV). Metoclopramide blocks dopamine D2 receptors in the central and peripheral nervous systems. This helps suppress PONV by reducing signals from the GI tract to the brain's vomiting center.^43^ These pharmacological interventions improve patient comfort and support earlier resumption of GI function. Thus, opioid-sparing analgesia and routine use of antiemetic agents are recommended to reduce postoperative GI dysfunction and enhance recovery after RC.

Early oral feeding is encouraged after RC, as studies have confirmed its safety and benefits for recovery. However, concerns remain in clinical practice about its potential link to anastomotic leakage and postoperative ileus.^44^ This traditional belief remains a significant barrier to implementing evidence-based recommendations, highlighting the gap between evidence and clinical practice regarding the advancement of oral nutrition in the postoperative period. Therefore, clinical efforts should focus on addressing misconceptions, enhancing patient and caregiver education, and promoting the early adoption of oral feeding protocols. Future research is warranted to determine the optimal timing and indications in RC patients.

Early mobilization is the core element of the ERAS framework. It has been shown to be associated with earlier GI function recovery and lower risk of postoperative ileus.^45^ Since most RC patients are elderly and have multiple comorbidities, surgery imposes a considerable physiological burden on them.^46^ Therefore, postoperative early mobilization is crucial to support recovery. Patients are encouraged to ambulate within 24 hours postoperatively, beginning with in-bed exercises, progressing to sitting or standing at the bedside, and finally achieving independent walking.^47^

This study incorporates TCM-based interventions, offering new perspectives for the PDG of RC patients. Among them, transcutaneous electrical acupoint stimulation (TEAS) is a modern adaptation of traditional acupuncture. It involves applying electrical stimulation to specific acupoints via surface electrodes to treat corresponding clinical conditions.^48^ TEAS may promote GI recovery by regulating the neuroendocrine network, particularly the brain–gut axis, thereby enhancing motility and accelerating the restoration of bowel function.^49^ TEAS has demonstrated favorable clinical outcomes in patients undergoing GI surgery. At the same time, its noninvasive nature further enhances both safety and patient acceptability.^5^^,^^50^ Although evidence regarding the use of TEAS in RC patients remains limited, the surgical trauma and postoperative GI recovery patterns are similar. The purpose and effects of the interventions are also aligned. Therefore, the evidence is considered highly generalizable and clinically applicable to the perioperative management of RC patients. Currently, there is no standardized protocol for TEAS parameters. Although most studies rely on the default settings of the stimulation device, there is consensus on using dense-disperse waveforms with a treatment duration of 30 minutes. The current intensity is usually tailored to each patient's tolerance level.^49^ Other TCM techniques, such as auricular therapy and acupressure, are also applied to support recovery. These approaches are based on the theory that specific health problems, such as depression, anxiety, and nausea, are reflected in specific acupoints located on the ear or other areas of the body. Stimulating these acupoints can help relieve related symptoms and support GI recovery.^51^ Therefore, TCM techniques may serve as valuable adjuncts to enhance postoperative GI recovery in RC patients, especially in settings where TCM resources and expertise are available. TEAS can be administered for 30 minutes per session, with the current intensity adjusted based on patient tolerance. For auricular therapy and acupressure, point selection should be tailored to specific symptoms such as nausea, anxiety, or delayed bowel motility. These approaches warrant broader application in the RC population.

### Implications for nursing practice and research

This study systematically summarized the best available evidence on perioperative GI management in RC patients, aiming to support the recovery of GI function after surgery. We recognize that the implementation of evidence must be grounded in clinical realities, patient preferences, and cultural values, while balancing its effectiveness, feasibility, and acceptability. We also incorporated evidence related to TCM techniques. However, promoting TCM-based interventions in predominantly Western nursing contexts remains challenging, due to cultural perceptions and differing standards for evaluating evidence applicability. Therefore, future practice should explore culturally adapted implementation strategies for TCM and develop integrative models that combine Eastern and Western nursing philosophies.

For clinical nursing practice, greater emphasis should be placed on enhancing nurses' evidence-based awareness and practical skills and strengthening multidisciplinary collaboration to translate best evidence into practice. From a research perspective, further empirical studies are needed to investigate barriers to implementation, cultural adaptability, and patient acceptance, thereby providing stronger theoretical and practical support for evidence-based nursing in multicultural contexts.

### Limitations

The limitation of this study is that only literature published in Chinese and English was included. Given the widespread use of TCM techniques in East Asian countries such as Japan and South Korea, relevant evidence published in other languages may have been missed due to language restrictions.

## Conclusions

This study conducted an evidence-based review on postoperative gastrointestinal function recovery in patients undergoing radical cystectomy, ultimately identifying 18 evidence-based recommendations across the three phases of the perioperative period. These recommendations address preoperative optimization, intraoperative protection, and postoperative interventions for supporting GI function. The findings provide scientific guidance for clinical practice. Future efforts should focus on generating original studies and implementation research to promote the application of these evidence in clinical settings and improve patient outcomes.

## CRediT authorship contribution statement

**Yumei Dai:** Conceptualization, Methodology, Formal analysis, Data Curation, Writing Original Draft; **Xiaoju Zhang:** Methodology, Writing - Review & Editing; **Qianwen Lin:** Methodology, Formal analysis; **Xiaofeng Gu:** Conceptualization, Resources, Supervision, Project administration, Writing - Review & Editing. All participants provided written informed consent.

## Ethics statement

Not required.

## Data availability statement

Data availability is not applicable to this article as no new data were created or analyzed in this study.

## Declaration of generative AI and AI-assisted technologies in the writing process

No AI tools/services were used during the preparation of this work.

## Funding

This study received no external funding.

## Declaration of competing interest

The authors declare no conflict of interest.
